# Online Information-Seeking Behavior Regarding Vitreous Floaters in the United States: Google Trends Analysis

**DOI:** 10.2196/94821

**Published:** 2026-10-13

**Authors:** Thanaphat Seeboonruang, Qais A Dihan, Jamie L Surgent-Nahay, Muhammad Z Chauhan, Ahmed F Shakarchi, Ahmed B Sallam

**Affiliations:** 1 Department of Ophthalmology, University of Arkansas for Medical Sciences, 4301 W Markham St, #523, Little Rock, AR, 72205, United States, 1 5016865822

**Keywords:** public interest, vitreous floaters, Google Trends, vitrectomy, demand index, digital ophthalmology

## Abstract

**Background:**

Vitreous floaters are a prevalent phenomenon, with some patients experiencing persistent symptoms that substantially degrade their vision. Public interest and information-seeking behavior regarding vitreous surgery are challenging to quantify using claims alone.

**Objective:**

The aim of this study is to evaluate temporal and geographic patterns of public interest in vitreous floaters and related interventions in the United States and to contextualize this interest relative to retina specialist availability.

**Methods:**

In this retrospective observational study, we queried Google Trends for search terms originating in the United States from 2008 to 2025. We extracted the relative search volume (RSV) using a composite of terms combined with the Google Trends “+” operator. State-level counts of vitreoretinal surgeons from the American Society of Retina Specialists (ASRS) database were used to calculate surgeon density per 100,000 population. We calculated a search-to-surgeon index (SSI) as the ratio of state-level RSV to retina surgeon density to serve as an exploratory metric of public interest relative to specialist availability. We evaluated associations using the Mann-Kendall trend test, Sen slope, and Spearman correlation.

**Results:**

From 2008 to 2025, nonsurgical RSV increased by 138%, demonstrating a significant temporal increasing trend (*τ*=0.600; *P*<.001). States with a higher proportion of residents aged 40 years or older demonstrated higher RSV (ρ=0.51; *P*<.01). The median SSI was 123 (IQR 100-127; range 48-311). Surgical RSV increased by 133% (*τ*=0.443; *P*<.001). The median surgical SSI was 133 (IQR 107-164; range 34-341).

**Conclusions:**

This analysis demonstrates substantial geographic variability in public interest in vitreous floaters across the United States. Integrating Google Trends data with surgeon distribution provides an exploratory framework for evaluating relative interest in vitreoretinal care, though it does not directly measure clinical demand.

## Introduction

Vitreous floaters are a prevalent phenomenon, with large community surveys suggesting that two-thirds of individuals notice floaters, and one-third perceive them as a visual impairment [1]. Most floaters are benign, but some patients may experience persistent symptoms that substantially degrade their vision and quality of life [2]. The true prevalence of symptomatic floaters requiring clinical intervention remains uncertain, but recent studies suggest a high burden in the population [3,4]. The increase in both aging populations and the incidence of myopia in the United States might cause an increase in public interest in floater-related surgical intervention. Public interest and information-seeking behavior regarding vitreous surgery for symptomatic floaters are challenging to quantify using claims or surgical volume alone, as these metrics reflect use rather than public awareness. Google Trends offers an option for quantifying real-time public interest. Accordingly, we used Google Trends to characterize temporal and regional variation in US public search interest for vitreous floaters and floater-directed interventions [5].

## Methods

### Ethical Considerations

This study did not use patient data but rather publicly available data on search engine trends in conjunction with publicly available US Census data. As such, no direct patient contact or intervention was involved, and individual informed consent was not required. Therefore, our study was exempted by the University of Arkansas for Medical Sciences Institutional Review Board. Our study was conducted in accordance with the Declaration of Helsinki.

### Google Trends Query Strategy

We queried Google Trends for searches originating in the United States between 2008 and 2025. Google Trends reports relative search volume (RSV) on a normalized scale from 0 to 100, with 100 representing peak popularity for a given search term for the selected time and geographical location. The searches were performed using a composite of search terms, which we grouped into 2 categories. We use the Google Trends “+” operator to combine exact syntax and capture public interest. The nonsurgical category included “floaters,” “eye floaters,” “cobwebs in vision,” “visual floaters,” and “floating specks.” The surgical category included “vitreous floaters,” “vitrectomy,” “symptomatic vitreous opacities,” “floaterectomy,” and “YAG vitreolysis.” Although “floaterectomy” is a colloquial and nonstandard medical term, it was intentionally included to capture niche public search variations that patients may use during online information seeking; the term is used to describe combined phacoemulsification and vitrectomy procedures for floaters [6]. The search terms were selected based on consensus among fellowship-trained vitreoretinal surgeons to capture both colloquial, symptom-based layperson queries and formal, treatment-based clinical inquiries. At the time of data collection (January 10, 2026), Google Trends allowed a maximum of 5 syntax inputs per query.

These terms reflect established clinical precedent, supported by previous studies evaluating vitreous floaters and prior evidence-based reviews that specifically use keywords such as “floaters,” “vitreous opacities,” “floaterectomy,” and “vitrectomy” in their search strategies [7,8].

### Retina Surgeon Density

We obtained state-level counts of vitreoretinal surgeons from the American Society of Retina Specialists (ASRS) member database [9]. We calculated retina surgeon density for each state by dividing the number of ASRS members by the 2025 US Census Bureau state population estimates and multiplying by 100,000 [10,11].

### Search-to-Surgeon Index

We calculated a search-to-surgeon index (SSI) to contextualize public interest relative to specialist availability [12,13]. We defined SSI as the ratio of state-level RSV to retina surgeon density (SSI=state-level RSV/retina surgeon density). Because RSV is a normalized, unitless scale rather than an absolute search count, the SSI is used strictly as an exploratory relative metric to rank geographic disparities rather than to quantify absolute clinical volume or health care demand.

### Population Age Distribution

Because Google Trends does not provide demographic data on users’ age, we independently obtained state-level population age distributions from the 2025 US Census Bureau. We used the proportion of residents aged 40 years or older as a proxy for populations at higher risk for symptomatic vitreous floaters. We evaluated the correlation between age distribution, RSV, and SSI using the Spearman correlation test.

### Statistical Analysis

We evaluated associations using the Mann-Kendall trend test with the Sen slope estimator and the Spearman correlation test performed using Microsoft Excel.

## Results

### State-Level Retina Specialist Availability and Population Age Structure

We identified 1992 US-based ASRS members. Mean retina surgeon density was 0.58 (SD 0.2; range 0.17-2.09) per 100,000 residents. Delaware (2.09), Maryland (1.04), and Vermont (0.93) had the highest densities, whereas Wyoming (0.17), Alaska (0.27), and New Hampshire (0.28) had the lowest. Census data showed the highest proportions of residents aged 40 years or older in Maine, Montana, New Hampshire, Pennsylvania, Vermont, and West Virginia, while the lowest proportions were in Alaska, Texas, Utah, Georgia, and North Dakota.

### Nonsurgical Floaters Search Trends

From 2008 to 2025, RSV for nonsurgical floater terms increased by 138%. Temporal trend analysis demonstrated a strong positive monotonic association between time and RSV values (Kendall *τ*=0.600; *P*<.001), with an approximate median increase of 0.172 RSV units per month (95% CI 0.153‐0.190). The minimum RSV of 42 occurred in November 2008 (Figure 1). There was a moderate positive correlation between the proportion of residents aged 40 years or older and RSV (ρ=0.45; *P*<.01). In 2025, the median RSV across states was 68 (IQR 61-73; range 48-100), with the highest values in Delaware (100), Alabama (77), and Tennessee (77) and the lowest in Utah (48), Wyoming (53), Montana (55), and Oregon (56) (Figure 2).

**Figure 1.**
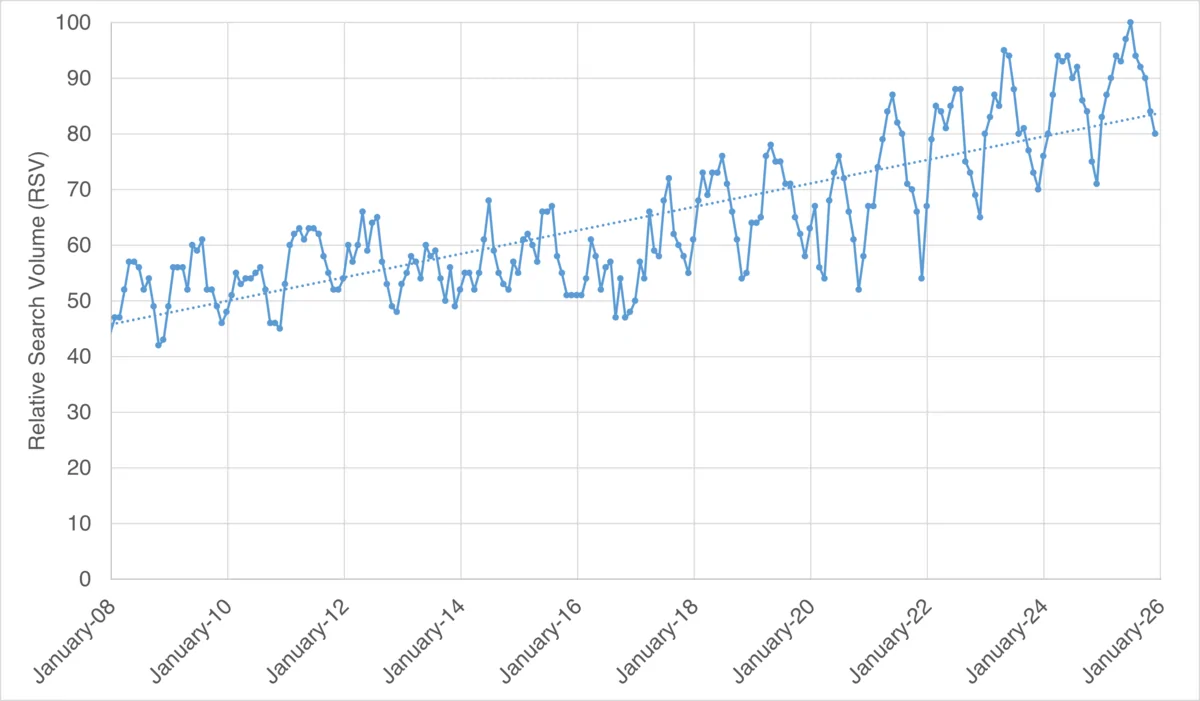
Trends in public interest in nonsurgical vitreous floaters over time. Time series of RSV for composite nonsurgical floater–related search terms from January 2008 to December 2025. RSV: relative search volume.

**Figure 2.**
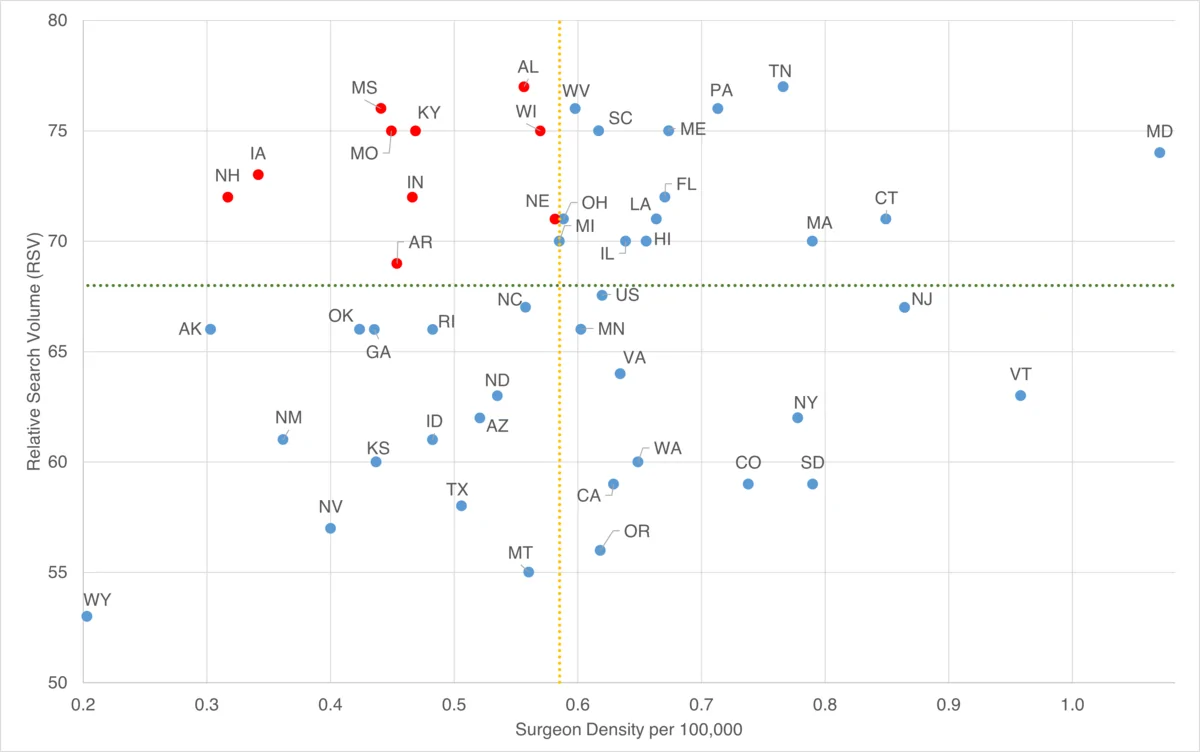
State-level relationship between nonsurgical public interest and surgeon density. Relationship between relative search density (RSV) for composite nonsurgical–related search terms and surgeon density per 100,000 people in 2025. Green dash: median RSV. Orange dash: median surgeon density. Red dot: state with RSV above the median and surgeon density below the median. Delaware (RSV 47.8; surgeon density 2.09) was excluded from the figure to enhance interpretability.

### Nonsurgical SSI

The median nonsurgical SSI across states was 123 (IQR 100-127; range 48-311). Wyoming (311), New Hampshire (254), and Alaska (244) had the highest SSI values, whereas Delaware (48), Vermont (68), and Maryland (71) had the lowest (Figure 3). There was no significant correlation between population age distribution and SSI (ρ=−0.08; *P*>.10).

**Figure 3.**
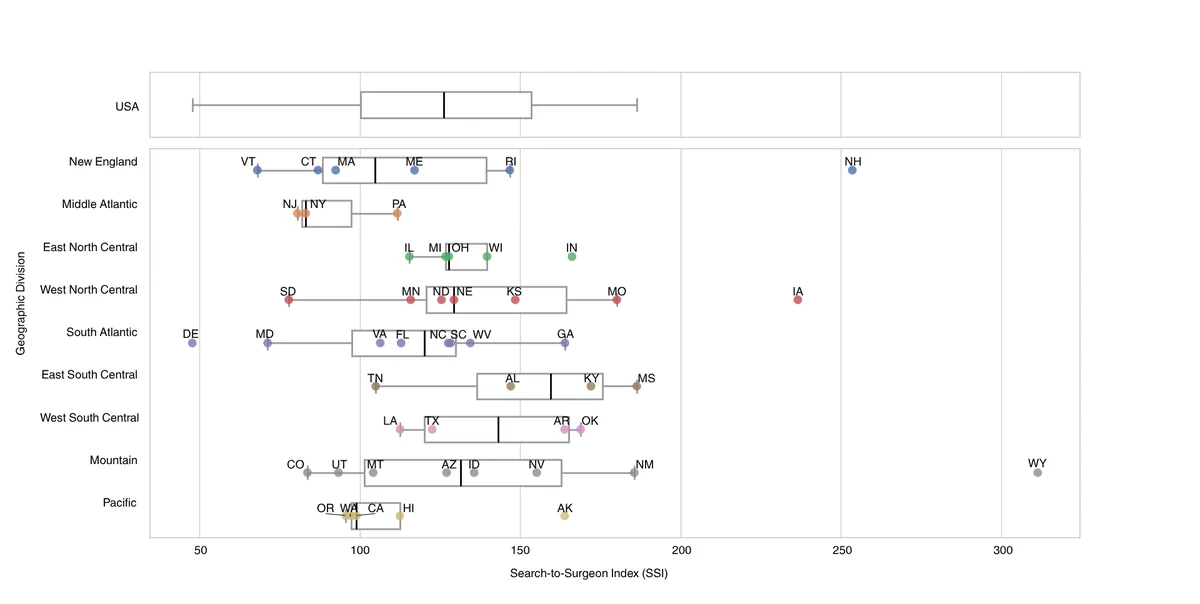
Geographic variation in the search-to-surgeon index (SSI) for nonsurgical floaters across the US. SSI for nonsurgical floaters across the US by Census Bureau geographic division. Overall US SSI is represented by the topmost box plot.

### Surgical Floater Search Trends

From 2008 to 2025, RSV increased by 133%. Temporal trend analysis demonstrated a moderate positive monotonic association between time and RSV values (Kendall *τ*=0.443; *P*<.001) with an approximate median increase of 0.101 RSV units per month (95% CI 0.084‐0.118) (Figure 4). In 2025, the median RSV across states was 70 (IQR 62-79; range 50-100). West Virginia (100), South Dakota (100), Montana (95), and Nebraska (95) demonstrated the highest RSV values, while Alaska (50), Georgia (58), California (58), Wyoming (58), and Mississippi (58) demonstrated the lowest (Figure 5). The proportion of residents aged 40 years and older showed a low but statistically significant positive correlation with surgical RSV (ρ=0.39; *P*<.01).

**Figure 4.**
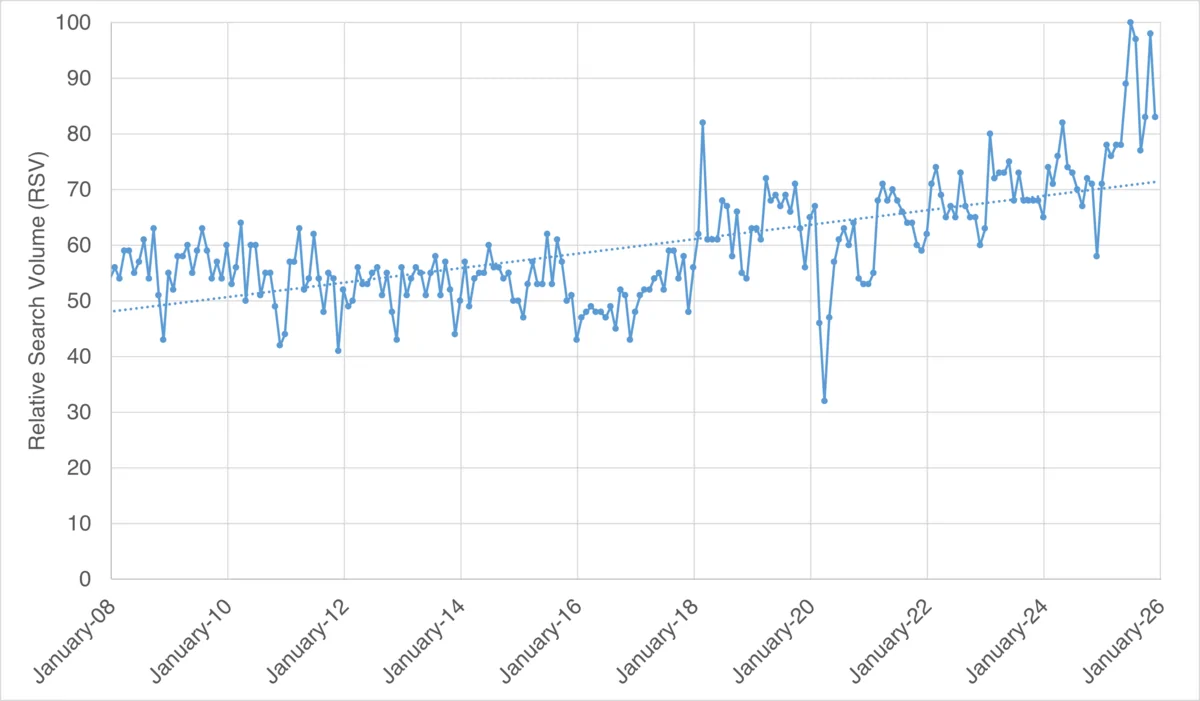
Trends in public interest in surgical vitreous floaters over time. Time series of RSV for composite surgical floater–related search terms from January 2008 to December 2025. RSV: relative search volume.

**Figure 5.**
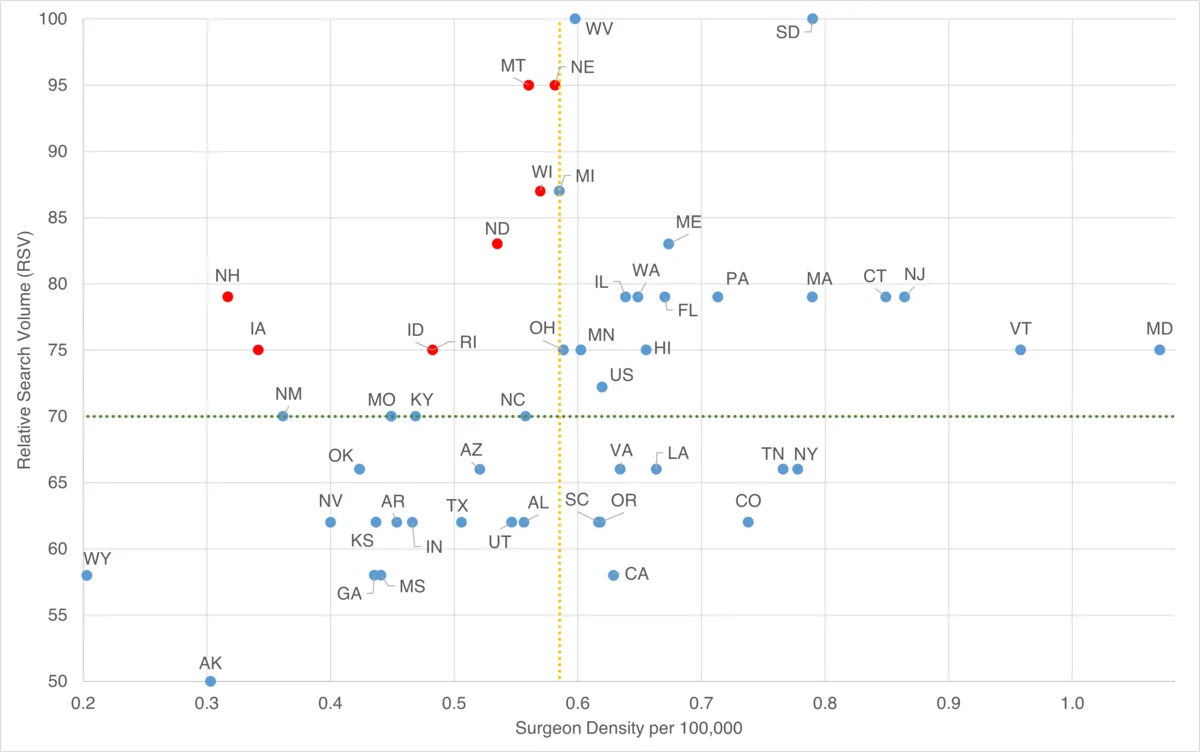
State-level relationship between surgical public interest and surgeon density. Relationship between relative search volume (RSV) for composite surgical–related search terms and surgeon density per 100,000 people in 2025. Green dash: median RSV. Orange dash: median surgeon density. Red dot: state with RSV above the median and surgeon density below the median. Delaware (RSV 33.5; surgeon density 2.09) was excluded from the figure to enhance interpretability.

### Surgical SSI

The median surgical SSI across states was 133 (IQR 107-164; range 34-341), with the highest values in Wyoming (341), New Hampshire (278), and Iowa (243) and the lowest in Delaware (34), Maryland (72), and Vermont (81) (Figure 6). Population age distribution did not significantly correlate with surgical SSI (ρ=−0.02; *P*>.10).

**Figure 6.**
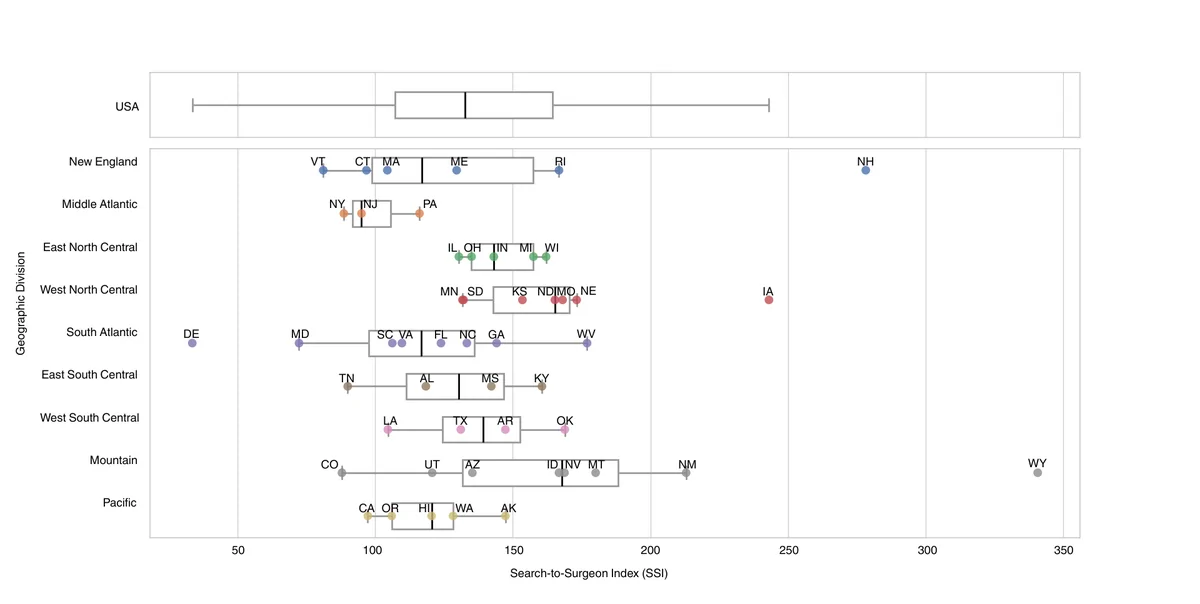
Geographic variation in the search-to-surgeon index (SSI) for surgical floaters across the US. SSI for surgical floaters across the US by Census Bureau geographic division. Overall US SSI is represented by the topmost box plot.

## Discussion

### Principal Findings

Public search interest in vitreous floaters and related interventions increased substantially across the US over the study period. These findings suggest symptomatic floaters are an increasingly important topic for online health information seeking.

Notably, our data demonstrated a significant upward inflection in RSV around March 2018, which coincided with the publication of a randomized clinical trial demonstrating improvement in floater symptoms after yttrium-aluminum-garnet (YAG) laser vitreolysis, and subsequent media coverage [14,15]. However, this correlation does not prove causality; multiple factors, including broader media coverage, social media activity, and changes in search engine algorithms, could have simultaneously influenced general search behavior. Similarly, a temporary decline in search growth in early 2020 coincided with the COVID-19 pandemic. While this mirrors reported reductions in elective ophthalmic procedures, it remains a speculative association without direct data on changes in individual health search patterns [16].

When public interest was interpreted relative to local specialist availability via the SSI, regional variations emerged. For instance, Delaware’s high surgeon density and SSI reflect its smaller population and proximity to major metropolitan referral centers, which act as cross-state catchments. Due to its extreme positive skew in surgeon density (2.09 per 100,000), Delaware was excluded from select visual analyses to preserve the interpretability of the scatterplots for the remaining states. Conversely, states with high SSI values and low surgeon densities such as Wyoming and Alaska may be indicative of regions where public interest outpaces local capacity, raising the possibility of regional disparities in subspecialty access [17,18].

### Limitations

Our study has several limitations. First, due to the inherent nature of internet search data, state-level RSV is an indirect indicator that cannot be used to infer individual-level care-seeking behavior, true clinical demand, or disease prevalence. Online search behavior is influenced by multiple confounding factors—such as educational campaigns, baseline internet access, and digital health literacy—which were not empirically adjusted for in this study. Furthermore, failure to adjust for state-level confounding variables, such as socioeconomic status, health insurance rates, and local referral patterns, increases the possibility of spurious relationships.

Second, our search strategy was constrained by Google Trends, which restricts exact-match term combinations (using the “+” operator) to a maximum of 5 inputs per query. While our composite categories used the 5 most representative terms determined by expert consensus, this platform limitation inherently omits less common and niche search variations, potentially underestimating the true totality of public interest. In the absence of published data on the exact usage rates of these search terms, our query strategy relied on expert consensus and historical literature precedent, which may not capture all relevant patient searches.

Third, using ASRS membership as a proxy for the total retinal workforce likely underestimates the true density of treating providers, as many patients with floater symptoms are initially evaluated by general ophthalmologists and optometrists. ASRS density is a crude proxy that does not account for an individual surgeon’s clinical capacity, surgical volume, or interstate referral dynamics.

Finally, without external validation against actual patient attendance data, referral rates, or surgical volume, the operational relevance of the SSI to workforce policy decisions remains strictly exploratory.

### Conclusions

Public search interest in vitreous floaters and related interventions has increased substantially across the United States from 2008 to 2025. Integrating Google Trends data with surgeon distribution provides an exploratory framework for evaluating relative public interest in vitreoretinal care. Future studies incorporating more granular workforce and referral data are required to validate whether these search patterns translate into measurable gaps in clinical access.

## Abbreviations

ASRS: American Society of Retina Specialists
RSV: relative search volume
SSI: search-to-surgeon index
YAG: yttrium-aluminum-garnet

## References

[1] Webb BF, Webb JR, Schroeder MC, North CS (2013). Prevalence of vitreous floaters in a community sample of smartphone users. Int J Ophthalmol.

[2] Albrecht M, Auffarth GU, Friedrich M, Kessler LJ, Khoramnia R (2025). Vision degrading myodesopsia from vitreous floaters in the young: an important aspect of myopia. Surv Ophthalmol.

[3] Lippek J, Rynko L, Framme C, Kermani O, Johannsmeier S, Ripken T (2025). Investigating symptomatic vitreous opacities: an online survey and field of view reconstruction. Klin Monbl Augenheilkd.

[4] Woudstra-de Jong JE, Manning-Charalampidou SS, Vingerling JHR, Gerbrandy SJF, Pesudovs K, Busschbach JJ (2025). The impact of vitreous floaters on quality of life: a qualitative study. J Patient Rep Outcomes.

[5] Cheong JH, Nayer ZH, Trief DF (2025). Demand for cataract surgery in the United States: Google Trends analysis. J Cataract Refract Surg.

[6] Mossa F, Delaney YM, Rosen PH, Rahman R (2002). Floaterectomy: combined phacoemulsification and deep anterior vitrectomy. J Cataract Refract Surg.

[7] Delaney YM, Oyinloye A, Benjamin L (2002). Nd:YAG vitreolysis and pars plana vitrectomy: surgical treatment for vitreous floaters. Eye (Lond).

[8] Ivanova T, Jalil A, Antoniou Y, Bishop PN, Vallejo-Garcia JL, Patton N (2016). Vitrectomy for primary symptomatic vitreous opacities: an evidence-based review. Eye (Lond).

[9] Find a retina specialist. American Society of Retina Specialists.

[10] Feng PW, Ahluwalia A, Feng H, Adelman RA (2020). National trends in the United States eye care workforce from 1995 to 2017. Am J Ophthalmol.

[11] State population by characteristics: 2020-2025. US Census Bureau.

[12] Lieu AC, Chuter BG, Radgoudarzi N (2024). Geographic patterns of ocular oncologist supply and patient demand for uveal melanoma treatment in the United States: a supply and demand analysis. Clin Ophthalmol.

[13] Doepker CP, Pakhchanian H, Raiker R, Lakhani DA, Hogg JP (2022). Google Trends data of radiologists who accept Medicare: a potential tool for predicting state demand. Curr Probl Diagn Radiol.

[14] Shah CP, Heier JS (2017). YAG laser vitreolysis vs sham YAG vitreolysis for symptomatic vitreous floaters: a randomized clinical trial. JAMA Ophthalmol.

[15] Stuart A, Sebag J, Shah CP, Gaurav SK (2018). To treat—or not to treat—vitreous floaters. EyeNet.

[16] Azad AD, Mishra K, Lee EB (2022). Impact of early COVID-19 pandemic on common ophthalmic procedures volumes: a US claims-based analysis. Ophthalmic Epidemiol.

[17] Pepper CM, Sandefer RH, Gray MJ (2010). Recruiting and retaining physicians in very rural areas. J Rural Health.

[18] Ahmed A, Ali M, Dun C, Cai CX, Makary MA, Woreta FA (2025). Geographic distribution of US ophthalmic surgical subspecialists. JAMA Ophthalmol.

